# Inflammation Meets Metabolic Disease: Gut Feeling Mediated by GLP-1

**DOI:** 10.3389/fimmu.2016.00154

**Published:** 2016-04-22

**Authors:** Tamara Zietek, Eva Rath

**Affiliations:** ^1^Department of Nutritional Physiology, Technische Universität München, Freising, Germany; ^2^Chair of Nutrition and Immunology, Technische Universität München, Freising, Germany

**Keywords:** incretins, ER stress, diabetes mellitus, microbiota, inflammation, enteroendocrine cells, glucagon-like peptide 1, inflammatory bowel disease

## Abstract

Chronic diseases, such as obesity and diabetes, cardiovascular, and inflammatory bowel diseases (IBD) share common features in their pathology. Metabolic disorders exhibit strong inflammatory underpinnings and vice versa, inflammation is associated with metabolic alterations. Next to cytokines and cellular stress pathways, such as the unfolded protein response (UPR), alterations in the enteroendocrine system are intersections of various pathologies. Enteroendocrine cells (EEC) have been studied extensively for their ability to regulate gastrointestinal motility, secretion, and insulin release by release of peptide hormones. In particular, the L-cell-derived incretin hormone glucagon-like peptide 1 (GLP-1) has gained enormous attention due to its insulinotropic action and relevance in the treatment of type 2 diabetes (T2D). Yet, accumulating data indicate a critical role for EEC and in particular for GLP-1 in metabolic adaptation and in orchestrating immune responses beyond blood glucose control. EEC sense the lamina propria and luminal environment, including the microbiota via receptors and transporters. Subsequently, mediating signals by secreting hormones and cytokines, EEC can be considered as integrators of metabolic and inflammatory signaling. This review focuses on L cell and GLP-1 functions in the context of metabolic and inflammatory diseases. The effects of incretin-based therapies on metabolism and immune system are discussed and the interrelation and common features of metabolic and immune-mediated disorders are highlighted. Moreover, it presents data on the impact of inflammation, in particular of IBD on EEC and discusses the potential role of the microbiota as link between nutrients, metabolism, immunity, and disease.

## Introduction

Metabolically driven pathologies, such as obesity, insulin resistance, and type 2 diabetes, but also immunologically mediated disorders, such as inflammatory bowel diseases (IBD), are considered chronic diseases. Concomitant with the spread of the western lifestyle, the prevalence of these diseases has rapidly increased (1–3) now constituting a global health problem (4). Even though phenotypically different, these diseases share common features in their pathology. Metabolic disorders exhibit strong inflammatory underpinnings and vice versa, inflammation is associated with metabolic alterations. For example, obesity evokes a broad array of inflammatory and metabolic responses leading to low-grade local inflammation and in turn to defective insulin receptor signaling and disruption of metabolic homeostasis (5). Conversely, patients suffering from sepsis and animal models of endotoxin-induced inflammation show a loss of glycemic control (6, 7). On a molecular level, metabolically driven and immunologically mediated disorders converge on cellular stress responses, such as endoplasmic reticulum unfolded protein response (ER UPR) (8). Furthermore, many chronic diseases are associated with increased levels of the pro-inflammatory cytokines tumor necrosis factor (TNF) and interleukin 6 (IL-6) (9, 10) as well as alterations in the intestinal microbiome (11, 12).

During the past years, the intestine and in particular, intestinal epithelial cells (IEC) are more and more recognized as key players in maintaining metabolic and immune homeostasis. Constituting a surface area up to 40 m^2^ (13) and being the bodies’ most important interface with the external environment, the intestinal epithelium needs to allow efficient nutrient absorption while maintaining barrier function and modulating immunity (12). Different epithelial cell subtypes fulfill distinct functions, with absorptive enterocytes and secretory (mucin-producing) goblet and (antimicrobial peptides-producing) Paneth cells accounting for the largest proportion of epithelial cells. Enteroendocrine cells (EEC) found scattered throughout the intestine comprise approximately only 1% of the epithelium but secrete more than 20 different peptide hormones, making them collectively one of the largest endocrine systems (14). Among the gut hormones, glucagon-like peptide 1 (GLP-1) and glucose-dependent insulinotropic polypetide (GIP), referred to as incretins, have gained enormous attention due to their insulinotropic action and relevance in the treatment of type 2 diabetes (T2D) (15). Especially GLP-1 and GLP-1-based antidiabetic therapies are in the spotlight of biomedical research. GLP-1 secreted by L cells not only potentiates the glucose-induced insulin response, promotes β-cell survival, slows gastric emptying (GE), and regulates energy expenditure and body weight, but also exerts neuroprotective, cardioprotective, and anti-inflammatory effects. Experimental approaches and treatment of patients with GLP-1 analogs as well as dipeptidyl peptidase-IV (DPP-4) inhibitors, which inhibit the endopeptidase that rapidly degrades GIP and GLP-1, underline the multiple beneficial effects mediated by GLP-1, beyond blood glucose control. While the mechanisms underlying hormone secretion from EEC and their functions in the context of digestion and metabolism are well studied, their role in intestinal inflammation and their interrelation with immune cells is virtually unknown. Recent data indicate that EEC, next to nutrients, also actively sense the microbiota and bacterial products, enlightening a new aspect of the cross-talk between immune and endocrine system.

This review illustrates L cell and GLP-1 functions and discusses the effects of incretin-based therapies on metabolic and inflammatory signaling. It focuses on the interrelation and common features of metabolic and immune-mediated disorders converging on GLP-1. Moreover, it presents data on the impact of intestinal inflammation, in particular of IBD, on EEC and the potential contribution of EEC to these pathologies. In addition, the possible roles of cytokines, UPR, and the microbiota as link between nutrients, metabolism, immunity, and disease are discussed.

## Enteroendocrine Cells: Luminal Sensing and L Cell Function

Typically, EEC have been classified based on the primary hormone they contain. EEC secreting GLP-1, GLP-2, and peptide YY (PYY) are referred to as L cells, whereas GIP-producing cells are considered K cells. However, it was demonstrated that expression patterns of L cells differ considerably depending on their location in the intestine (16–18). In the proximal small intestine, L cells also express considerable amounts of GIP, cholecystokinin (CCK), and secretin.

**Figure 1:**
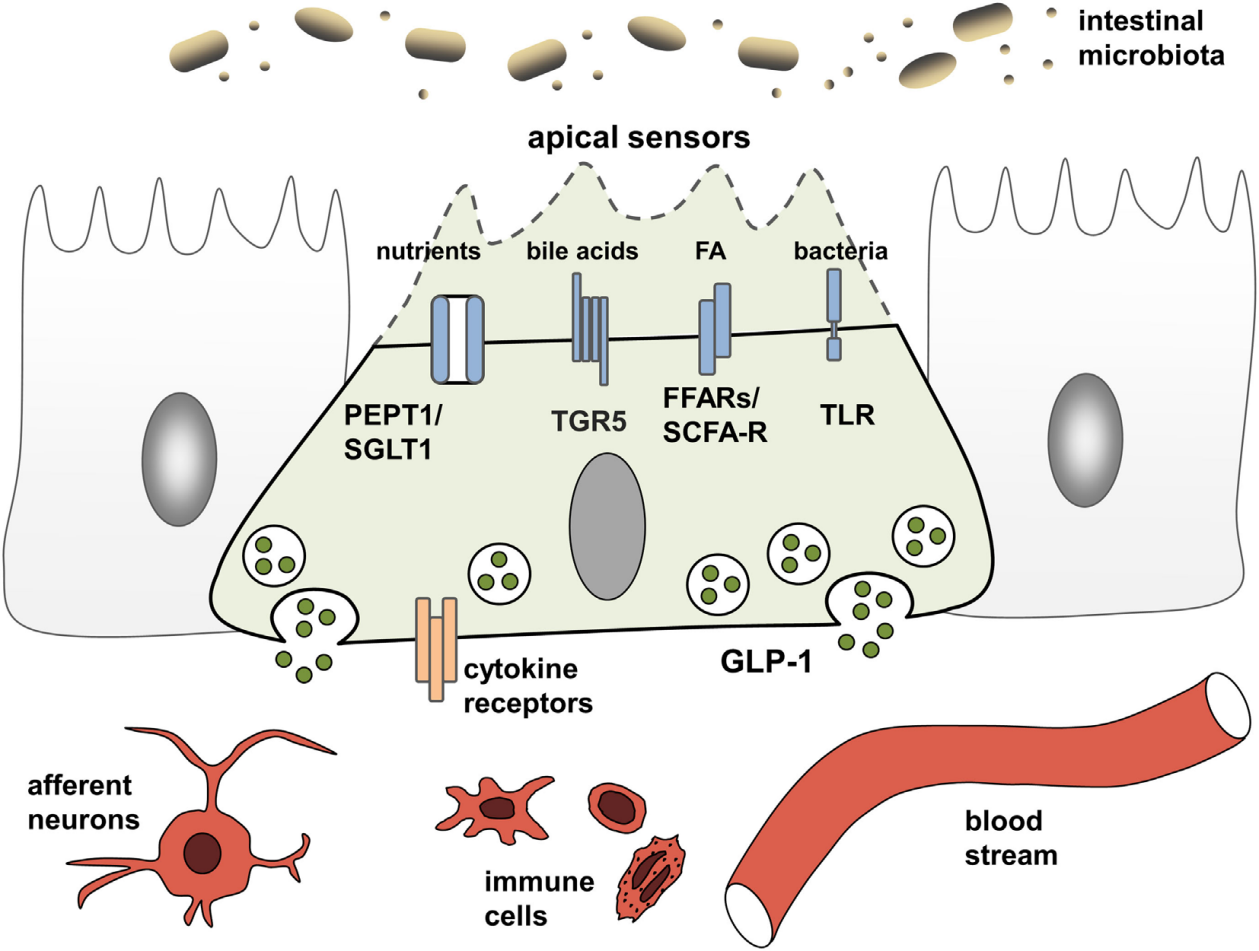
**L cells as interface between luminal-derived signals and host metabolism and immune system**. L cells sense the luminal and lamina propria environment via receptors and transporters and mediate signals through hormone and cytokine secretion. Fatty acids (FA), free fatty acid receptor (FFAR), short-chain fatty acid (SCFA), sodium-dependent glucose transporter (SGLT1), proton-coupled peptide-transporter (PEPT1), glucagon-like peptide 1 (GLP-1), G-protein-coupled bile acid receptor 1 (TGR5).

EEC have been studied extensively for their ability to adjust gastrointestinal motility, secretion, and insulin release thereby enabling efficient postprandial assimilation of nutrients (19). To achieve this prime function of creating the optimal absorptive and digestive conditions following nutrient intake, EEC are equipped with specific apical sensor proteins for the detection of distinct luminal nutrients. Depending on the location along the gut axis, luminal stimuli of EEC comprise monosaccharides, free fatty acids (FA), monoacylglycerols, amino acids, di/tripeptides bile acids, short-chain fatty acids (SCFAs), and indole. The following sections briefly summarize receptors and transporters involved in environmental sensing by L cells (Figure 1). For more detailed information, see Ref. (15, 19).

### Nutrient Sensing

Carbohydrates, lipids, and proteins are detected via their digestion end products. Luminal glucose represents the most potent secretagogue for incretin hormone release *in vivo*, an effect mainly mediated by the sodium-dependent glucose transporter, SGLT1 (SLC5A1) (15). As indicated by experiments demonstrating concomitant ingestion of proteins and glucose to enhanced GLP-1 responses (20), peptides and amino acids are sensed by EEC via the proton-coupled peptide-transporter PEPT1 (SLC15A1) and the calcium-sensing receptor (CaSR) (15). In the case of SGLT1 and PEPT1, nutrient-sensing is linked to hormone secretion by the electrogenic character of the transporters. Substrate transport is coupled to cation influx causing membrane depolarization, subsequent opening of voltage-gated calcium channels and an increase in intracellular calcium levels, finally resulting in exocytosis and hormone release from vesicles (15). This sensing property of nutrient transporters in EEC has coined the term “transceptors” and has extended the group of sensor proteins. Before SGLT1 was identified as the first transceptor, only G-protein-coupled receptors (GPCR) were known to fulfill such sensory functions in the intestine. GPCR comprise the amino acid- and oligopeptide-sensing receptors CaSR (15), metabotropic glutamate receptors (20), LPAR5/GPR92/93 (21), GPRC6A (22), and taste receptors (TRs) (23). Furthermore, GPCR are responsible for detection of lipid-derived long-chain/medium-chain FA and dietary-fiber derived SCFAs. Predominantly, lipid ingestion causes GIP release. Yet, FA also elicit GLP-1 responses via the free fatty acid receptors (FFAR) FFAR1/GPR40, FFAR4/GPR120, and GPR119. Dietary fibers, after fermentation to SCFAs by the microbiota, are sensed by FFAR2/GPR43 and FFAR3/GPR41 and also trigger GLP-1 secretion (24).

Along the lines of GLP-1 analogs and DPP-4 inhibitors, various agonists of GPR40 and GPR120 have been developed and proven effective in ameliorating glycemic control in animal trials (21, 22), now being in phase I or II clinical trials for the treatment of T2D (23). Interestingly, high fat consumption is associated with enhanced L cell density in obese subjects and mice (25). This increase in the number GLP-1-positive cells observed in mice was attributed to improved L cell differentiation and, furthermore, SCFAs were sufficient to increase L cell numbers in intestinal organoids of human and murine origin (25, 26). It has been suggested that this effect, favoring insulin secretion, might constitute an adaptive response of the intestine to counterbalance diet-induced insulin resistance.

Several other transporters and receptors, such as the facilitative glucose transporter (GLUT2), are thought to play a role in incretin secretion, however, their contribution to luminal compound sensing remains unclear.

### Taste Receptors

Taste receptors detecting complex tastes, such as sweet, umami, and bitter taste, are present in the intestinal epithelium including EEC. While taste 1 receptors function as dimers to sense sweet or umami, taste 2 receptors detect a large variety of bitter tastants (26). Consequently, tastants might induce or contribute to the release of peptide hormones from EEC (24, 27). Studies indicate a physiological role for umami (TAS1R1/R3) and bitter TRs, yet, the relevance of the sweet TR T1R2/T1R3 is highly controversial (15, 19, 27). T1R2/T1R3 is activated not only by sugar but also by non-metabolizable sweeteners, such as saccharin (28). Although studies using EEC lines and tissue explants have demonstrated GLP-1 release in response to T1R2/T1R3 ligands, the *in vivo* relevance of sweet TR activation on incretin secretion still remains unclear, since others have been unable to demonstrate functional activity of T1R2/T1R3 in primary cultured L cells or in perfused intestinal preparations (15, 19). In line, *in vivo* studies in animals and humans consistently failed to show effects of artificial sweeteners on plasma incretins (28–30). However, sweet TR activation results in increased apical SGLT1 levels and, via this effect, might contribute to incretin secretion (15). Interestingly, a selective upregulation of the bitter TR TR2R138 was shown in the colon of mice fed a high fat diet (31), and T2R38, a human receptor activated by the same ligand, phenylthiocarbaminde, has been demonstrated not only to be expressed in EEC of the colonic mucosa but also to be induced in overweight/obese subjects (32). T2R38 is known to respond to Gram-negative bacterial quorum-sensing molecules in human upper airway cilia thereby regulating innate immune responses (33). It is attractive to speculate that these receptors function as sensors for subpopulation of the intestinal microbiota and might respond to the alterations of gut microbial communities associated with long-term high-fat diet and obesity.

### Pattern Recognition and Sensing of Bacterial Products

While a role for T2Rs in microbial sensing of EEC still needs to be addressed, there is clear evidence that EEC respond to bacteria and bacterial products. In particular, EEC possess functional toll-like receptors (TLR) and upon lipopolysaccharide (LPS) stimulation, GLP-1 release is triggered in mice (34). Also, bacterial metabolites, such as SCFA and indole, a product of bacterial tryptophan metabolism involved in interbacterial communication, exert direct signaling actions on colonic L cells (19). Further evidence for the importance of the microbiota for incretin regulation comes from germ-free (GF) and antibiotic-treated mice, which have severely reduced SCFA levels, and concomitantly increased basal GLP-1 plasma levels as well as increased proglucagon expression, specifically in the colon (35). Increasing energy supply suppressed proglucagon expression in GF mice, suggesting that colonic L cells sense energy availability and regulate basal GLP-1 secretion accordingly.

Next to indole, which acts on voltage-gated K^+^ channels to enhance Ca^2+^ entry thereby stimulating GLP-1 secretion (36) and microbiota-derived SCFA that are sensed by FFAR2/GPR43 and FFAR3/GPR41 (see above), the importance of bile acid-induced incretin secretion via the bile acid receptor GPBAR/TGR5 has been proven *in vitro* and *in vivo*. TGR5 ligands improve insulin sensitivity and glucose homeostasis through the secretion of incretins (27) and the rapid improvement of hyperglycemia after bariatric surgery has been attributed partly to alterations in bile acid metabolism and sensing (29, 30). A central role of bile acid signaling as a novel pharmacological target in the metabolic syndrome and related diseases, such as obesity, T2D, atherosclerosis, liver disease, and cancer, is underscored by the presence of TGR5 in various tissues and cell types. Activation of TGR5 in brown adipose tissue and skeletal muscle increases energy expenditure, while activation in macrophages inhibits production of pro-inflammatory cytokines (37).

Through affecting bile acid metabolism and providing SCFA, the intestinal microbiota might indirectly impact TGR5-signaling and nutrient sensing leading to incretin hormone secrection, yet, directly triggering activation of EEC via TLR also evokes an inflammatory response (38). Upon exposure to flagellin or LPS (39, 40) EEC express pro-inflammatory cytokines. TLR activation evokes a specific gene response indicating EEC to participate in innate immune responses to commensals and pathogens (39). Together with observations that immune cells are in close physical contact with EEC (41), this led to the suggestion of an immunoendocrine axis and a critical role for EEC in orchestrating intestinal immune responses (42).

In the context of bacterial pattern recognition, it is noteworthy that PEPT1, known to mediate di/tripeptide-induced GLP-1 secretion, was also shown to transport small bacterial-derived peptides, such as muramyl dipeptide (MDP). Intracellularly, these peptides can be sensed by nucleotide-binding oligomerization domain (NOD)-like receptors (NLR) that belong to the innate immune system and recognize pathogen-associated molecular patterns (43). Yet, there is neither data on the transport and possible consequences of bacterial-derived proteins in EEC nor on the expression of NLR in EEC. However, *Pept1^−/−^* mice do not show any abnormalities in weight or any other anthropometric or clinical chemistry measurement when animals are fed a standard high-carbohydrate diet (44). Furthermore, the role of PEPT1 in intestinal inflammation remains controversial, since there is conflicting data on expression levels under inflammatory conditions in mice and humans (43, 45, 46).

## GLP-1 Actions

### GLP-1 Effect on Blood Glucose Control

Upon stimulation, L cells secrete different peptide hormones, including the incretin GLP-1. GLP-1 is derived from a transcription product of the proglucagon gene *gcg*, which also encodes GLP-2 and further factors. Mainly expressed in the ileum and colon, GLP-1 is known as the most powerful incretin in humans and lowers postprandial blood glucose via augmentation of glucose-dependent insulin release from pancreatic β-cells, inhibition of glucagon secretion from pancreatic α-cells, and delay of GE. Furthermore, GLP-1 increases pancreatic β-cell growth by promoting proliferation and reducing apoptosis, an effect that might be predominantly mediated locally via α-cell-derived GLP-1 (47). Conversely, L cells are responsive to insulin and insulin resistance is associated with impaired GLP-1 secretion *in vitro* and *in vivo* (48). These properties constitute the basis for GLP-1-based antidiabetic therapies, yet GLP-1 also exerts anorexigenic effects by promoting satiety and reducing food intake.

Glucagon-like peptide 1 and GIP act via G-protein-coupled receptors. The GLP-1R is expressed in many tissues, including pancreatic islets, the central nervous system, lung, kidney, heart, intestine, and also on immune cells (49, 50), underlining the numerous roles for GLP-1-signaling beyond blood glucose control.

When secreted by L cells, GLP-1 either functions in an endocrine manner, being released into the blood stream where it is rapidly inactivated by DPP-4 with a half-life of about 2 min, or exerts paracrine effects like stimulating neurons. Triggering vagal afferents, GLP-1 mediates signaling from gut to brain with anorexigenic effects and via nerve terminals in the hepatoportal region, it can affect metabolic functions in the liver (15). In line, peptide hormones secreted by EEC can modulate immune responses via the nervous system. For instance, is has been shown that nutritional stimulation of CCK receptors attenuated inflammation through inhibition of pro-inflammatory cytokine secretion from macrophages via the vagus nerve (51).

### GLP-1 and Immune System

There is a strong cross-talk between immune and endocrine system (Figure 1). Data indicate that immune cells and cytokine mediated-signaling impacts EEC numbers during infection and chronic inflammation of the gut (52, 53). On the other hand, there is a widespread expression of GLP-1R on multiple immune cell populations, particularly on intestinal intraepithelial lymphocytes (IEL) (50, 54). Moreover, in addition to its enzymatic functions, DPP-4 is known as the lymphocyte cell surface protein CD26, which plays a key role in T-cell development, activation, and immune regulation (55, 56). Last but not least, insulin itself was shown to inhibit IL-10-mediated regulatory T-cell functions (57) and also GLP-1 exerts direct anti-inflammatory effects. Supporting a function of GLP-1 in modulating immune responses, circulating GLP-1 was reported to be increased in states of chronic inflammatory disease, including the metabolic syndrome, coronary artery disease, or heart failure (58–60) but also in critically ill patients and patients with sepsis (61). Furthermore, treatment with GLP-1 as well as GLP-1 analogs and DPP-4-inhibitors exhibit beneficial effect in a vast array of diseases.

## Incretin-Based Therapies

GLP-1-based therapies and DPP-4-inhibition, which also targets GIP by increasing its half-life, have been proven successful in the treatment of T2D.

Common adverse effects of incretin mimetics and DPP-4 inhibitors include gastrointestinal symptoms, such as nausea and vomiting as well as hypersensitivity reactions. Safety issues have also been raised on a possible association between incretin-based drugs and pancreatitis or pancreatic cancer based on animal studies and data on patients (62). However, animal as well as patient data are inconsistent and suffer from several confounders (63, 64). Since the relationship remains unclear, both the Endocrinologic and Metabolic Drugs Advisory Committee and the FDA’s Division of Metabolism and Endocrinology Products concluded that there is not sufficient evidence available to conclude that incretin-based therapies cause acute pancreatitis or pancreatic cancer (65). Nonetheless, pancreatitis is continued to be considered a risk associated with these drugs until more data are available (65).

**Figure 2:**
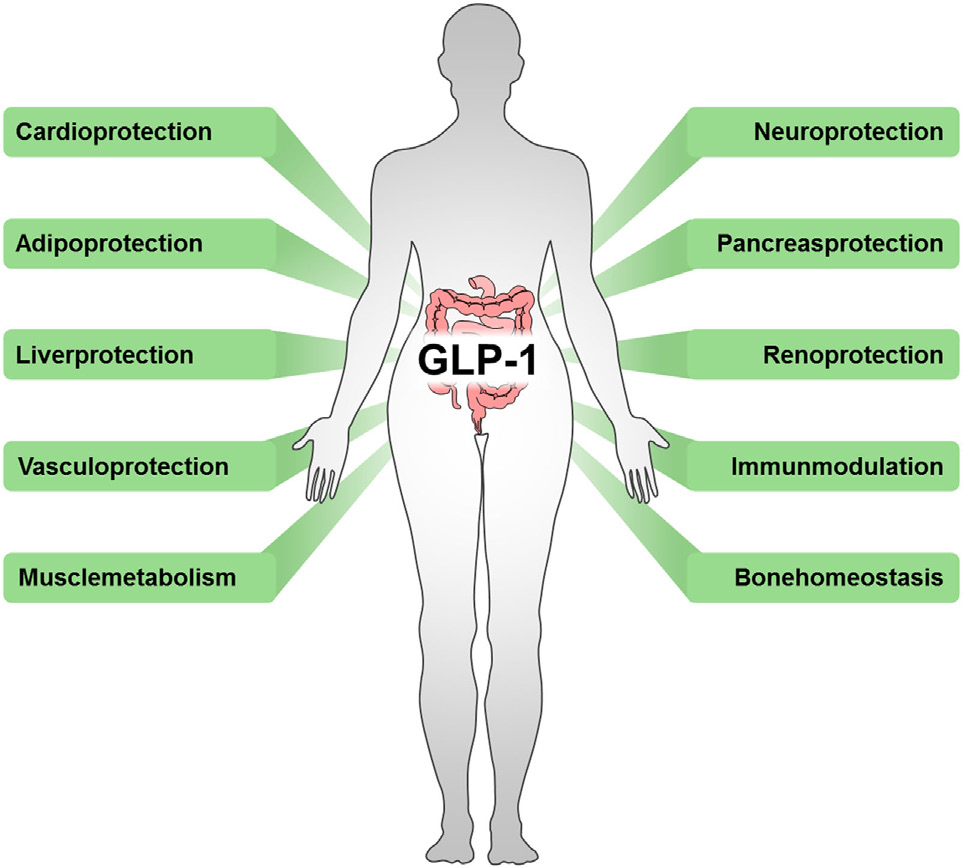
**Beneficial outcomes of drugs targeting incretins**. Incretin-based therapeutics, such as GLP-1 analogs, GLP-1R agonists, or DPP-4 inhibitors, exert multiple effects in various tissues.

In addition, several effects of incretin-based diabetes therapies beyond their glycemic-lowering properties via the endocrine pancreas have been described, including reduced immune cell infiltration and altered cytokine expression (66). Again emphasizing the interrelation of metabolic and inflammatory disorders, beneficial outcomes have also been observed in cardiovascular disease (67), stroke (68), hepatic diseases (69, 70), nephritis (71), neuro-inflammation (72, 73), rheumatoid arthritis (74), lung inflammation (75), and sepsis (7) (Figure 2).

### GLP-1 and GLP-1 Analogs

In patients with T2D, GLP-1 and GLP-1 analogs were shown to improve cardiovascular-risk profiles, by reducing body fat content, blood pressure, circulating lipids, and inflammatory markers (76). Furthermore, low-grade inflammation of the endothelium is an early event in the pathogenesis of atherosclerotic cardiovascular disease and the GLP-1 analog liraglutide was shown to exhibited anti-oxidative and anti-inflammatory effects, including upregulation of anti-oxidative enzymes and inhibition of nuclear factor kappa B (NF-κB)-signaling on endothelial cells (67, 77). Through inactivation of NF-κB, a master regulator of inflammatory responses, GLP-1 treatment of mice also significantly alleviated lung inflammation and pulmonary fibrosis (75). In mouse models of Alzheimer’s disease synthetic incretin hormones exerted neuroprotective effects, including reduced amyloid plaque load, reducing oxidative stress and the chronic inflammatory response in the brain, and enhancing neurogenesis (72, 73). In line, the GLP-1 receptor agonist Exendin-4 was suggested to down-regulate pro-inflammatory responses and reduce oxidative stress by suppressing MAPK signaling pathways in peripheral lymphocytes of patients with T2D (78). In addition, Exendin-4 was demonstrated to selectively reduce the production of cytokines from activated IEL, indicating a local enteroendocrine–immune axis (50). In an obese mouse model of diabetes, GLP-1 was shown to reduce macrophage infiltration and directly inhibit inflammatory pathways in adipocytes and adipose tissue macrophages (79). Notably, studies have shown beneficial effects of GLP-1 treatment on glycemia in critically ill patients and patients suffering from septic shock, a phenomenon also seen in patients with T2D. Concomitantly, the incretin effect was reduced in critically ill patients resembling previous findings in patients with T2D (6). In line, experimental evidence indicates beneficial effects of DPP-4-inhibition and GLP-1 analog treatment during sepsis. The improved survival of animals with LPS-induced endotoxemia by treatment with GLP-1 analogs (7) or endotoxin-challenged DPP-4 knockout rats (61) is associated with reduced vascular inflammation/dysfunction. In this context, the diminished levels of oxidative stress and tissue protection were attributed to direct anti-inflammatory capacities of GLP-1. In non-alcoholic fatty liver disease and hepatocyte steatosis, GLP-1 and incretin mimetics have been shown to ameliorate pathology by promoting macroautophagy and SIRT1-mediated signals (69, 70, 80).

### DPP-4 Inhibitors

DPP-4 activity is markedly increased in obese subjects and in animal models of obesity (81, 82), suggesting the endopeptidase as target for therapeutic interventions. DPP-4 inhibitors increase the amount of circulating GLP-1 and GIP by delaying their inactivation. On immune cells, inhibition of DPP-4/CD26 results in anti-inflammatory effects via regulation of chemokines and anti-inflammatory cytokines like IL-10 and transforming growth factor (TGF)-β (83, 84) and DPP-4 might also impact macrophage polarization. CD26 is furthermore involved in a variety of human autoimmune diseases, and as a cell surface protease, DPP-4/CD26 plays an important role in tumor progression (85). However, the majority of actions ascribed to DPP-4 in immune cells are attributable to non-enzymatic actions of the enzyme; hence, DPP-4 signaling in immune cells seems independent of its catalytic enzyme activity (86).

Nonetheless, DPP-4 inhibitors improve cardiovascular outcomes by modulating innate and adaptive immunity and suppression of the NLRP3 inflammasome, a multiprotein complex involved in caspase-1 activation and downstream maturation of pro-inflammatory cytokines, TLR4 and IL-1β in human macrophages (87, 88). For ischemic stroke and retinal damage, two major complications of diabetes mellitus, it was shown that DPP-4 inhibition prevented inflammation and mediated neuroprotective effects (68, 89). These properties were attributed to antioxidant, anti-inflammatory, and anti-apoptotic mechanisms, including suppression of NF-κB and downstream of the inflammatory cytokines TNF and IL-6. Concomitantly, the anti-inflammatory cytokine IL-10 was found to be elevated (68). In addition, data indicate that DPP-4 inhibitors also impact immune cell recruitment and reduced macrophage infiltration seemed to be mediated directly via GLP-1-dependent signaling in a rat nephritis model (71).

In summary, these observations underline the tight interrelation of endocrine signaling and immune responses and, furthermore, indicate common disease mechanism for metabolic and immune-mediated disorders.

## Common Features of Metabolic and Immune-Mediated Disorders Impacting GLP-1

### Immune Mediators/Cytokines

**Figure 3:**
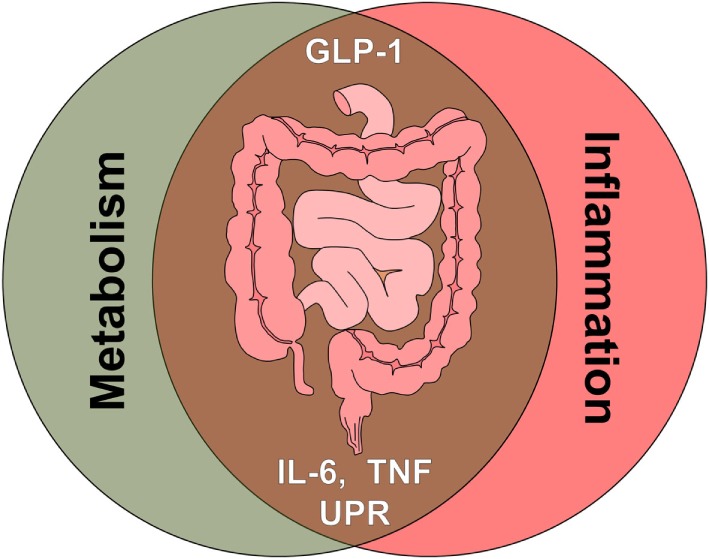
**The intestine as crossroad of metabolism and inflammation**. Multiple signals associated with metabolic signaling and inflammatory signaling, such as gut hormones, cytokines, and cellular stress pathways, converge at the level of the intestine. Glucagon-like peptide 1 (GLP-1), tumor necrosis factor (TNF), interleukin 6 (IL-6), unfolded protein response (UPR).

Yet another strong link between metabolic and immune-mediated disorders are elevated levels of the cytokines TNF and IL-6, underlying several pathologies and both being reported to impact on GLP-1 signaling (Figure 3).

#### Tumor Necrosis Factor

Both TNF and IL-6 are correlated with the body mass index (BMI), and TNF is known to play a role in obesity and in particular in the insulin resistance and diabetes that often accompany obesity (10, 90). Typically, TNF is involved in systemic inflammation and the acute phase reaction. It is able to induce fever, apoptotic cell death, cachexia, inflammation, and to inhibit tumorgenesis. Besides obesity, dysregulation of TNF production has been implicated in a variety of human diseases, including Alzheimer’s disease (91), cancer (92), rheumatoid arthritis (74), and IBD (93). Critically ill patients demonstrate elevated levels of TNF and IL-6 and as mentioned above, these patients and T2D share phenotypical similarities such as hyperglycemia, insulin resistance, and systemic inflammation (6). During obesity, pro-inflammatory macrophages accumulate in adipose tissue representing a chronic low-grade inflammation; these cells are the dominant sources of TNF promoting insulin resistance (9). Neutralization of TNF improves glucose uptake in murine obesity and mice lacking TNF are protected from high-fat-diet-induced insulin resistance (10, 94). L cells express TNF receptor 1 (TNFR1) and chronic exposure to TNF was shown to impair GLP-1 (95) as well as GLP-2 secretion (96). In mice with high-fat diet-induced hyperglycemia, hyperinsulinemia, and associated induction of TNF, a reduction of GLP-1-secretion was observed, which could be reversed by treatment with the TNF-neutralizing antibody etanercept (95). In a human study, infusion of TNF-induced systemic inflammation and concomitantly reduced plasma levels of GLP-1 (97).

Tumor necrosis factor is a therapeutic target in several diseases and anti-TNF treatment is successfully applied in rheumatoid arthritis, psoriasis, ankylosing spondylitis, and IBD. Considering the effects of TNF on L cells and data from animal models, indicating that impaired glucose tolerance following high-fat diet-induced obesity can be ameliorated by anti-TNF therapy (95), one might expect a significant impact of anti-TNF treatment on glucose metabolism. However, inhibition of TNF does not alter the state of insulin resistance in IBD patients (98, 99), and no differences in plasma insulin, glucose, and insulin resistance were noted in pediatric Crohn’s disease (CD) patients when comparing pre- and post infliximab (a chimeric anti-TNF antibody) treatment measurements (100). Furthermore, long-term therapy with etanercept did not alter fasting GIP-1 levels in patients with active rheumatoid arthritis. No changes were observed in these patients before and after anti-TNF treatment in the plasma GLP-1-response to an oral glucose challenge (101). In summary, these data underline that there is no simple correlation between TNF, GLP-1, and glycemic control under physiological conditions, but an interrelated disease network, including numerous factors.

#### Interleukin 6

Next to TNF, the cytokine IL-6 possesses the best-documented impact on incretin hormone secretion. IL-6 exerts pro-inflammatory as well as anti-inflammatory effects depending on its source and the context of secretion. T cells, macrophages, adipocytes, and myocytes are among the cells secreting IL-6. As a pro-inflammatory cytokine, IL-6 participates in the acute phase reaction and stimulates immune responses, especially during infection and tissue damage (102). In this context, inhibition of the IL-6 pathway was successfully applied in the treatment of rheumatoid arthritis (103). The anti-inflammatory properties comprise inhibition of TNF and IL-1 as well as activation of IL-10 (102). Within the intestine, IL-6 has been shown to prevent epithelial apoptosis and promote epithelial proliferation during injury and inflammation (104). IL-6 is also produced in muscle, with increased levels following muscle contraction. During exercise, it is thought to act in a hormone-like manner to stimulate energy mobilization, leading to increased body temperature and highlighting its metabolic functions (102). Obesity and T2D are associated with elevated plasma concentrations of IL-6, with adipose tissue being the major source under these conditions (105, 106). Several studies demonstrated IL-6 to regulate glucose tolerance and insulin action (107, 108) and furthermore, IL-6 was shown to enhance insulin secretion by increasing GLP-1 production in L cells and alpha cells, leading to improved glucose tolerance (47). Hence, IL-6 was suggested to mediate crosstalk between insulin-sensitive tissues, intestinal L cells, and pancreatic islets through GLP-1. In the CNS, IL-1 and IL-6 were demonstrated to mediate GLP-1 receptor-induced suppression of food intake, and body weight (109). Additionally, significant associations of GLP-1 were found with markers of inflammation, including IL-6 and C-reactive protein (CRP) in critically ill patients, and endotoxin, IL-1, and IL-6 were sufficient to induce GLP-1 secretion in mice (61). Conversely, endotoxin-dependent hyperinsulinemia was markedly blunted by the use of GLP-1 receptor antagonists or in IL-6 knockout mice (61).

Several other cytokines and mechanisms have been implicated at the interface of metabolism and inflammation. For example, IL-10 has been shown to prevent diet-induced insulin resistance (110), and IL-6 and TNF are known to decrease levels of the protective adipokine adiponectin (111). RANTES, a pro-inflammatory chemokine, was demonstrated to reduce glucose-dependent secretion of GLP-1 and GLP-2, thereby impairing glucose-induced insulin secretion in mice (112).

### ER Unfolded Protein Response

A cellular condition on which metabolically mediated and inflammation-driven pathologies converge is ER stress and the associated UPR. Underlying various diseases, such as neurodegenerative disorders, diabetes, cancer, atherosclerosis, and IBD, the ER UPR has gained enormous attention during the past years (8). In mammalian cells, the ER is essential for cholesterol production, for calcium homeostasis, and for the transit of correctly folded proteins to the extracellular space. Hence, a functional ER UPR is essential to all secretory cells. Among the conditions that challenge ER functions and elicit ER stress responses are changes in calcium homeostasis or redox status, elevated protein synthesis, accumulation of unfolded or misfolded proteins, energy deficiency, and microbial infections (113). The purpose of the ER UPR is to restore ER homeostasis by enhancing protein degradation, reducing protein synthesis and expanding the protein folding capacity by upregulation of chaperones that help proteins in the ER lumen to fold (114). However, if the ER stress is prolonged or excessive, ER UPR can ultimately lead to cell death via apoptotic pathways (115, 116).

Inflammation and ER stress are linked at many levels; inflammation is characterized by the production of large amounts of proteins, such as cytokines or chemokines and, furthermore, studies using mice deficient in ER UPR-mediators link ER stress in the highly secretory subtypes of IEC, antimicrobial peptides-producing Paneth cells and mucin-producing goblet cells, with antimicrobial defense and intestinal inflammation (117–119). In addition, ER UPR-signaling can directly intersect with inflammatory pathways, including NF-κB, TLR-mediated signaling, and production of reactive oxygen species (ROS) (120–123). Yet, ER UPR is involved not only in inflammatory responses but also in metabolic processes and adipocyte differentiation (124, 125).

It has been suggested that the ER is essential in the coordination of metabolic responses through its ability to control the synthetic and catabolic pathways of various nutrients (126). These features are reflected by the responsiveness of the ER UPR to the nutritional state of mammalian cells (127). ER UPR impacts glucose as well as lipid metabolism (8). Consequently, there is strong evidence that ER UPR plays a critical role in pancreatic β cell survival and is relevant in the pathology of diabetes, obesity, and insulin resistance. Pancreatic β cells need to dramatically increase their insulin production to fit the demand under chronic insulin resistance and, hence, pancreatic islets from mice and humans with T2D show signs of ER UPR (128). Several factors associated with obesity and T2D, such as inflammatory cytokines and free FA, can induce ER UPR (129, 130). In particular, ER UPR-mediated activation of JNK has been linked to the development of insulin resistance and diabetes by inhibition of insulin receptor signaling (131); and various animal models bearing modifications in ER UPR-associated functions demonstrate defects in pancreatic β cells and impaired glucose metabolism (131–134).

Notably, several chemicals used to treat T2D like PPAR agonists or salicylates have been shown to affect ER UPR-associated pathways (135, 136). In murine models of obesity and diabetes, administration of the chemical chaperones phenyl butyric acid (PBA) and tauro-ursodeoxycholic acid (TUDCA), increased systemic insulin sensitivity, established normoglycemia, reduced fatty liver disease, and suppressed inflammatory signaling (137). PBA and TUDCA were furthermore shown to prevent ER stress-induced inhibition of apoB100 secretion, a feature contributing to hepatic steatosis (138) and to ameliorate atherosclerosis in mouse models (139). In the context of IBD, oral administration of either PBA or TUDCA reduced the severity of dextran sulfate sodium (DSS)-induced acute colitis as well as chronic colitis in IL-10-deficient mice (140). During intestinal inflammation, the loss of secretory Paneth and goblet cell is a well-described phenomenon. This effect has been linked to ER UPR-signaling (118, 119, 141), and ER stress has also been demonstrated to counteract epithelial stemness (142). Interestingly, intestinal L cells have been reported to be prone to gluco- and lipotoxicity, with lipotoxicity being associated with activated ER stress response (143). Yet, there are virtually no reports on ER stress, ER UPR, and the functional consequences in L cells under disease conditions. However, it has been shown that GLP-1 analogs reduce hepatocyte steatosis and improve survival by enhancing UPR and promoting macroautophagy (69), and that postoperative increases in circulating cholic acid concentration contribute to improvements in glucose homeostasis after IT surgery by ameliorating ER stress (144). In conclusion, ER stress and the associated signaling might be a promising target for further research and future therapeutic interventions on the level of EEC.

## Intestinal Inflammation and Enteroendocrine Cells

**Figure 4:**
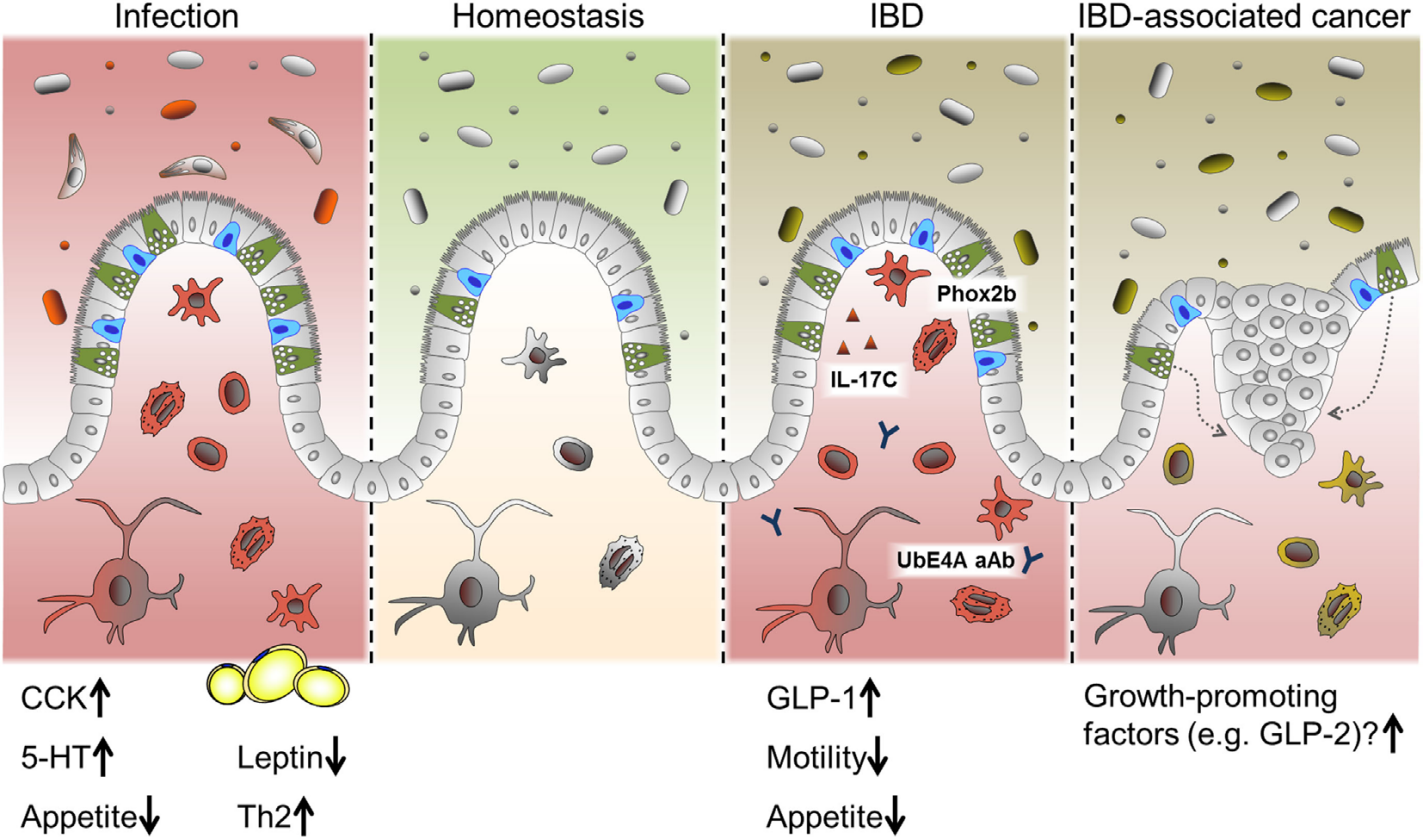
**EEC in intestinal pathologies**. Alterations in EEC and EEC-derived hormones have been reported under conditions of acute intestinal inflammation such as infections and chronic intestinal inflammation like IBD. While CCK and 5-HT are predominantly induced during infection, several roles for EEC are proposed in IBD. Suggested mechanism include a SNIP in the EEC-associated transcription factor Phox2b, auto-antibodies (aAB) against the EEC protein UbE4A, IL-17C secretion and increased levels of GLP-1. Changes in gut motility and appetite are also reported during intestinal inflammation. Long-standing inflammation like in persistent IBD is associated with an increased risk of developing carcinomas. Growth-promoting factors produced by EEC might contribute to this progression.

Intestinal epithelial cells are crucial for maintaining intestinal homeostasis, constituting an interface between the two major factors influencing intestinal inflammation, the gut microbiota and the immune system (12). IEC directly sense enteric luminal bacteria and interact with immune cells of the lamina propria as well as IELs and are, therefore, considered to be a constitutive component of the mucosal immune system (12, 145). As mentioned above, alterations in IEC subpopulations have been described previously in the context of intestinal inflammation, in particular for mucin-producing goblet cell and antimicrobial-peptide-producing Paneth cells. Yet, alterations in EEC numbers and secretion of gut hormones have also been observed in intestinal inflammation and were implicated in the changes in feeding patterns often accompanying these conditions (146, 147) (Figure 4).

### Enteroendocrine Cells during Infection

In intestinal infection, responses on the level of EEC seem to be a conserved mechanism throughout different species and infectious agents. In this context, CCK-positive cells appear to be a main target of regulation, and increases in this cell type have been reported in fish, lamb, pigs, mice, and humans during infection (42, 53, 148). Interestingly, hyperplasia of selected EEC subtypes in response to intestinal infection is probably dependent on immune cells, since serotonin (5-HT)-producing cell hyperplasia associated with *Citrobacter rodentium* infection was absent in mice lacking adaptive immunity (53) and CD4^+^ T cells were necessary to increase numbers of 5-HT producing enterochromaffin cells during helminth infection (149). Of note, increased levels of CCK are correlated with the period of hypophagia seen during enteritis. In an elegant study, Worthington et al. demonstrated that the reduction of fat mass and the resulting decrease in the adipokine leptin due to hyperphagia, was necessary for an effective Th2 immune response and, subsequently, efficient parasite expulsion (146). Intriguingly, in human lymphocytic colitis, a condition with unclear etiology and characterized by accumulation of lymphocytes in the colonic epithelium and lamina propria, both chromogranin A (CgA) and PYY-positive cell densities were reported to be increased (150, 151). These results illustrate a different layer of the interaction of EEC/gut hormones, metabolism, and immunmodulation.

### Enteroendocrine Cells in IBD

Inflammatory bowel diseases and its two main idiopathic pathologies ulcerative colitis (UC) and CD are chronic, immunologically mediated disorders of the gastrointestinal tract. They are characterized by relapsing inflammations of the colon (UC) and the whole gastrointestinal tract (CD), respectively. UC as well as CD are multifactorial diseases and are associated with alterations of the innate and adaptive immune system, luminal and mucosa-associated microbiota as well as epithelial function (3). A failure to control inflammatory processes at the IEC level may critically contribute to IBD pathogenesis. So far, EEC have not attracted much interest in the research on IBD pathology. Yet, a gene wide association study (GWAS) has identified a single nuclear polymorphism (SNIP) in the enteroendocrine-associated transcription factor Phox2b as risk factor for Crohn’s disease (152) and auto-antibodies to the enteroendocrine protein UbE4A are associated with disease behavior (153). Utilizing double immunofluorescence techniques, a co-localization of Phox2b with CgA and GLP-1 was demonstrated (154). In this context, it is noteworthy that EEC have been observed to be a specific target of immune responses resulting in EEC depletion during acute small intestinal allograft rejection (155). Moreover, EEC have recently been identified as producers of IL-17C in IBD, a novel member of the IL-17 cytokine family, and most likely involved in the pathogenesis of active IBD (156).

Patients suffering from IBD often display metabolic changes concomitantly to altered adipokine levels and increased inflammatory parameters (157–160). While leptin level was shown to be inversely related to disease activity, resistin level was increased in patients with active disease and was identified as independent predictor of disease activity in CD (157, 160, 161). Insulin resistance and changes in lipid metabolism are a common phenomenon in IBD. Of note, the relative insulin resistance observed is in most cases due to increased insulin levels, whereas serum glucose remains normal (158–160, 162). Interestingly, Valentini et al. reported hyperinsulinemia in IBD patients to be associated with a decrease in adiponectin and proved hyperinsulinemia to be an independent protective factor for 6-month maintenance of remission (160). Intestinal inflammation is associated with elevated levels of circulating free FA in IBD. It has been suggested that these alterations reflect an “energy appeal reaction” of the organism providing free energy in the circulation, which is needed by inflammatory cells (158). Besides, reduced appetite, anorexia and altered intestinal motility often accompany intestinal inflammation, and might be linked to EEC, since PYY, GLP-1, and CCK signal satiety and modify motility (14, 163, 164).

Increased serum levels of human pancreatic polypeptide (HPP), gastrin, motilin, CCK, PYY, ghrelin, and also GIP, GLP-1, and GLP-2 have been reported either at baseline or postprandially in UC and/or CD (154, 163, 165–168). Several of these studies show conflicting results, which might be partly due to technical advancements, since some of the results have been published more than 30 years ago. However, some alterations in serum levels were reflected by changes in EEC-subtype numbers found by immunohistochemistry, substantiating these observations (154, 166, 169). Overall, the total number of studies and patients is limited, especially when regarding the heterogeneity of IBD pathologies. Yet, an increase in total EEC numbers defined as CgA-positive cells has been reported for ileal CD (154, 169). Furthermore underlining the link between EEC and chronic intestinal inflammation, T-cell receptor α knockout mice displaying reduced cytokine levels and decreased EEC numbers, develop an UC-like phenotype (52). For GLP-1, elevated levels were found in CD (165) and UC (167), whereas no differences were found in CD specifically affecting the small bowel (163). Noteworthy, Moran et al. showed increased numbers of GLP-1 and CgA-positive cells in terminal ileal CD, which was confined to the site of active inflammation. Neither in the presence of active colitis (without ileal involvement) nor in quiescent ileitis, EEC numbers were changed in the terminal ileum (154). This might indicate total changes in hormone secretion to be too minor to be recovered in serum due to the limited tissue affected. Yet, the changes in EEC numbers and activity observed seemed to be cell-type specific, since PYY expression remained unaltered, suggesting a selective induction of GLP-1 rather than a simple global upregulation of all EEC lineages and their products (154). On the other hand, DPP-4, the incretin degrading endopeptidase, has been found to be reduced in tissue and plasma in active CD (170) and further inhibition of DPP-4 accelerated mucosal healing in a murine model of colitis (171). Locally increased GLP-1 levels might exert paracrine functions on IEL (50) and afferent neurons, impacting mucosal immune responses and feeding patterns, respectively. Appetite is reduced in active small bowel CD, and in a subset of patients with IBD, GE is delayed, and prolonged GE is associated with higher disease activity and increased secretion of GLP-1. Conversely, GE is accelerated and GLP-1 release decreases significantly, following effective therapy (164).

The upregulation of GLP-1 positive cells might be linked to an increase in GLP-2, an L-cell-derived peptide and a further splice product of the *gcg* gene. GLP-2 is an epithelial growth factor implicated in epithelial homeostasis, barrier function, and repair following injury. GLP-2 exerts anti-inflammatory functions, such as sustaining proper Paneth cell function (172), and has been shown to ameliorate experimental colitis in several animal models (173–176). More important, a GLP-2 analog was proven effective in the treatment of active moderate to severe CD (177) and improved intestinal functions in patients with short bowel syndrome (178, 179).

In the context of epithelial homeostasis and wound healing, it is remarkable that incretin gene expression is regulated through the Wnt signaling pathway (180, 181). Wnt signals determine the intestinal stem cell niche and regulate intestinal stemness and proliferation (182). Chronic and acute intestinal inflammation is associated with alterations in cell proliferation most likely due to tissue insults and IEC loss. GLP-1 itself is capable of stimulating the Wnt pathway in an autocrine manner (183), suggesting a further potential mechanism linking incretins to intestinal tissue homeostasis.

On the other hand, clinical data enlighten further aspects of EEC function and metabolism. Surgical resection of affected parts of the intestine and colectomy are part of IBD treatment. Ileostomists show a significant reduction in circulating GLP-1 associated with elevated glucagon concentrations, indicating the colonic endocrine tissue to have an important role in postprandial metabolism (184). Enlightening a different aspect of the role of EEC under pathological conditions, long-standing intestinal inflammation is associated with an increased risk of developing adenocarcinomas. It has been suggested that an increased EEC mass in response to chronic inflammatory injury drives neoplasia by producing trophic hormones (185).

**Figure 5:**
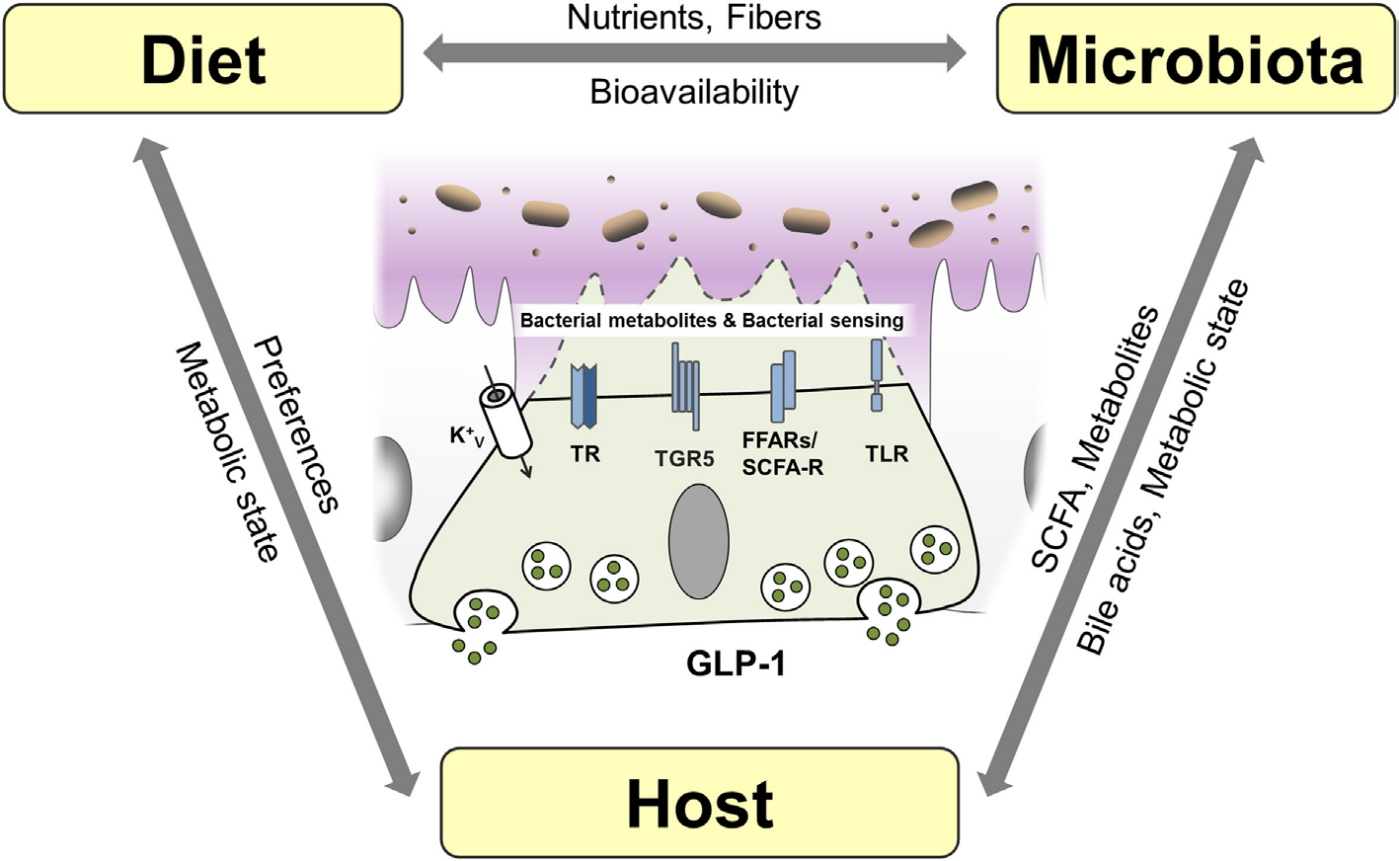
**Interaction of host, diet, and microbiota and proposed mechanisms of bacterial sensing in EEC**. EEC are able sense bacterial products such as short-chain fatty acids (SCFA) and the bacterial metabolite indole as well as bacteria through taste receptors (TR), free fatty acid receptors (FFAR)/SCFA receptors and K^+^ channels. Bile acids are sensed by EEC via TGR5 and can be metabolized and conjugated by the microbiota impacting host metabolism. Diet, microbiota and host genetic and metabolic makeup are interrelated factors influencing EEC function.

Enteroendocrine cells alterations rather seem to be a general feature of intestinal inflammation than a specific mechanism of IBD. To clarify if these alterations represent only a consequence or causatively contribute to intestinal inflammation, more research is needed. Yet, EEC and their products might modulate IBD pathology through orchestrating a metabolic-inflammatory response (Figures 1 and 5).

## Microbiota and Enteroendocrine Cells

In recent years, the intestinal microbiota and its correlation with numerous human pathologies has gained increasing attention. There is a complex interaction between host genetic and metabolic makeup, diet, and microbiota (Figure 5). Changes in terms of diversity and richness have been associated with conditions, such as obesity, T2D, Parkinson’s disease, cardiovascular disease, and IBD. Manipulating the microbial composition is, therefore, an attractive therapeutic approach; probiotics as well as prebiotics have been demonstrated to exert multiple beneficial effects in IBD (186) and T2D (187), including increased GLP-1 release (188). Concurrently, evidence from studies with GF mice indicates a huge impact of the microbiota on EEC numbers (189). More specifically, Glp1r knockout mice display an altered composition of their intestinal microbiota (50). As described above, EEC can directly sense their microbial environment via TLR and through metabolites generated the microbiota. Regarding GLP-1 expression, SCFA and bile acids are the best-studied links between microbiota and gut hormone secretion.

Short-chain fatty acids are produced by bacteria in the distal gut by fermentation of fibers and exert various beneficial effects on human health (190). In particular, butyrate has been shown to ameliorate mucosal inflammation and oxidative status, improving intestinal barrier, preventing colorectal cancer and beyond these intestinal effects, to reduce food intake, improve hypercholesterolemia, insulin resistance, and ischemic stroke (191). SCFA are able to modulate cytokine and chemokine expression of immune cells and adipocytes (190). They are sensed by L cells via FFAR2/GPR43 and FFAR3/GPR41, and activation of these receptors triggers GLP-1 secretion (15). The importance of SCFA-mediated GLP-1 secretion is highlighted by the observation that FFAR2 knockout mice exhibit reduced insulin levels and impaired glucose tolerance due to reduced GLP-1 levels (24). Furthermore, butyrate-induced GLP-1 secretion is attenuated in FFAR3 knockout mice (51). Interestingly, it was demonstrated that different compositions of the intestinal microbiota via their distinct abilities to produce SCFA might in turn affect expression of FFAR by epigenetic gene regulation (192). Conversely, modulating the composition of the microbiota using the probiotic VLS#3, stimulated production of butyrate, promoting GLP-1 secretion, and improving the metabolic state in mice (193). Of note, VSL#3 has also been shown to be effective in the treatment of IBD (186).

There is only limited data on the role of specific bacterial species on incretins; however, in an elegant study, Everard et al. showed that the abundance of *Akkermansia muciniphila* was associated with L cell number and secretory capacity (194). *A. muciniphila* is a mucin-degrading, acetate and propionate-producing bacterium residing in the mucus layer, thus being in relative close proximity to the intestinal epithelial layer (195), hence having the potential capacity to activate GPR43. Interestingly, *A. muciniphila* has been shown to be reduced in obese and T2D mice and that administration of *A. muciniphila* for 4 weeks was sufficient to reverse high-fat diet-induced obesity and T2D (196).

In contrast to SCFA, bile acids are produced by the host. Synthetized from cholesterol in the liver, bile acids facilitate formation of micelles in the intestine, promoting absorption of dietary fat (197), but additionally, they are increasingly recognized to act as signaling molecules and receptor ligands (198). The hepatic bile acid synthesis is tightly regulated by negative feedback mechanisms (199) and by transforming primary into secondary bile acid species, the intestinal microbiota strongly affects bile acid metabolism (200). Thus, modulating the bioavailability and resorption of bile acids, the microbiota impacts on enterohepatic bile acid-signaling and greatly impacts the whole-body metabolic homeostasis (199). Next to the bile acid receptor TGR5, which is directly linked to GLP-1 secretion from L cells, the farnesoid X receptor (FXR) was implicated in bile acid-induced metabolic alterations. Activation of FXR by the gut microbiota was shown to reduce the expression levels of most bile acid synthesis enzymes and inhibit the expression of gluconeogenic genes (201, 202), thereby linking the intestinal microbiota to the regulation of bile acids and metabolic homeostasis. Conversely, bile acids impact the bacterial composition of the microbiota by exhibiting strong antimicrobial functions (203).

Bile-acid sequestrants are used to sequester bile acids in the intestine, leading to increased bile acid synthesis and consequently to a reduction in low-density lipoprotein cholesterol. In patients with T2D, bile-acid sequestrants have furthermore been shown to improve glucose control. This effect was suggested to be due to multiple mechanisms, including alteration of the bile acid pool, changes in microbiota composition, improvement of hepatic glucose metabolism, and increased release of incretin hormones (204, 205). Also, some of the remarkable metabolic effects of bariatric surgery have been attributed to altered bile acid signaling (199). In line, TGR5 and FXR agonists have shown promising results in the treatment of metabolic and inflammatory diseases, such as IBD and T2D (37). Modulating the microbiota/SCFA/bile acid-signaling to impact metabolism and inflammatory processes via GLP-1 and other mechanisms, therefore, represents a new strategy for the treatment of several chronic diseases.

## Conclusion and Perspective

Exploring the role of EEC and incretin hormones beyond blood glucose control is still in its infancy. In the context of inflammation, incretins might be a double-edged sword, since peptide hormones secreted by EEC not only exert direct immunomodulatory effects on diverse immune cell subsets (14) but GIP signaling has also been associated with pro-inflammatory processes and insulin resistance in mice (206). The complexity of the immune–endocrine axis is further highlighted by the unexpected finding that mice overexpressing TNF are protected from high-fat diet-induced insulin resistance (207). Along this line, IL-6 knockout mice only display an altered metabolic phenotype when fed a high-fat diet (208). To tackle the question on the role of EEC under inflammatory conditions, in particular if and how they are involved in the onset of diseases, more research is needed.

**Figure 6:**
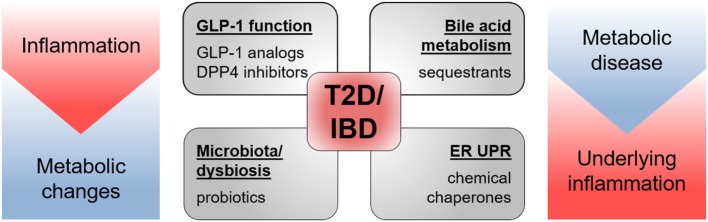
**Drugs targeting T2D and IBD pathology**. Drugs shown to be effective in animal models of and/or in patients with T2D as well as IBD target GLP-1 function, bile acid metabolism, microbiota, and endoplasmic reticulum unfolded protein response (ER UPR). Both pathologies share metabolic changes and inflammatory processes in their etiology.

Multiple drugs targeting GLP-1 functions, ER UPR, bile acid signaling, and the microbiota have already proven to be effective in the treatment of T2D and IBD (Figure 6). Yet, there seem to be much more possibilities and fields of applications for these therapeutics and more still to be developed. Promising new experimental tools and models, such as intestinal organoids (209), will allow elucidating remaining questions on EEC functions and may permit identification of new therapeutic and nutriceutical targets.

## Author Contributions

All authors listed, have made substantial, direct, and intellectual contribution to the work, and approved it for publication.

## Conflict of Interest Statement

The authors declare that the research was conducted in the absence of any commercial or financial relationships that could be construed as a potential conflict of interest.
